# Examining the role of IgA in a persistent model of *Staphylococcus aureus* colonization

**DOI:** 10.1371/journal.ppat.1013924

**Published:** 2026-01-28

**Authors:** Yunys Perez-Betancourt, Miaomiao Shi, Dominique Missiakas

**Affiliations:** Department of Microbiology, The University of Chicago, Chicago, Illinois, United States of America; Trinity College Dublin, IRELAND

## Abstract

*Staphylococcus aureus* is a human-adapted pathogen that replicates by asymptomatically colonizing its host. Nasal colonization occurs in the first weeks of life and persists in about 30% of the population. Using the mouse-adapted strain WU1 to model persistent colonization, we reported earlier that inoculation of bacteria lacking Staphylococcal protein A (SpA/Δ*spa*) or neutralization of SpA through vaccination result in the slow decolonization of animals. Secretory (S)IgA is considered a first line of defense against pathogens at mucosal surfaces. Here, we use *Ighasec*^-/-^ mutant mice to evaluate the contribution of SIgA towards decolonization. We observe that WU1 burdens are reduced in colonized *Ighasec*^-/-^ mice compared to C57BL/6J animals. Both C57BL/6J and *Ighasec*^-/-^ mice eliminate Δ*spa* bacteria, yet elimination occurs more rapidly in animals lacking IgA. SpA captures Fab-V_H_3-type antibodies, including IgA, on the bacterial cell surface. We propose that this activity promotes colonization. Yet, we also find that antibody responses to the pathogen are altered when SpA and IgA are missing. Colonized C57BL/6J mice display a low serum IgG2c/IgG1 ratio towards staphylococcal antigens. This ratio is increased in animals colonized with Δ*spa* and is further enhanced in *Ighasec*^-/-^ mice. We attribute the former to the loss of immune evasion activity in absence of SpA, and the latter to a host compensatory mechanism upon exposure to *S. aureus*. Importantly, the increased IgG2c/IgG1 ratio correlates with decolonization and enhanced killing of *S. aureus*. Similarly, we observe that decolonization induced by SpA-vaccination is accelerated in *Ighasec*^-/-^ mice which display higher anti-SpA IgG2c titers as compared to C57BL/6J animals. Together, these findings suggest that *S. aureus* exploits SIgA in a SpA-dependent manner for colonization and in absence of IgA, serum opsonizing antibodies may promote bacterial clearance at mucosal surfaces.

## Author summary

The mucosa is constantly exposed to microbes keeping it clear of pathogens while tolerating those beneficial to the host. Secretory immunoglobulin A (SIgA) contributes to such homeostasis by inhibiting microbial attachment, neutralizing toxins, and controlling inflammatory responses at mucosal surfaces. Yet, *Staphylococcus aureus*, a deadly pathogen, colonizes the upper respiratory track of humans asymptomatically. This behavior suggests that *S. aureus* subverts mucosal immune responses. Here, we compare *S. aureus* colonization in wild type and mice lacking IgA. We find that while colonization is not strictly impaired, bacterial burdens in IgA deficient animals are reduced. Staphylococcal protein A (SpA), a protein that coats the bacterial surface and binds antibodies takes advantage of IgA for colonization. In absence of bacterial SpA or host IgA, animals develop a high ratio of IgG2c/IgG1 against *S. aureus* indicative of a Th1 response*.* In absence of both SpA and IgA, this response is further expanded and correlates with a slow but steady loss of *S. aureus* carriage. In a vaccination approach aimed at neutralizing the immune suppressive activities of SpA, animals lacking IgA undergo accelerated decolonization compared to wild type animals. These findings suggest that *S. aureus* exploits SIgA during colonization, SIgA antibodies are not required for vaccine-mediated decolonization, and serum opsonizing antibodies elicited by the Th1 response may promote bacterial clearance at mucosal surfaces.

## Introduction

*Staphylococcus aureus* (*S. aureus*) is a major human pathogen responsible for a broad spectrum of diseases, from superficial skin infections to life-threatening conditions such as pneumonia, sepsis, and endocarditis [1–3]. A distinguishing feature of *S. aureus* is its ability to persist in the human nasopharynx. Epidemiological studies indicate that *S. aureus* colonization occurs at birth and is either persistent (~ 30% of individuals) or intermittent (30–50% of the human population) [4–8]. Colonization facilitates infection and the risk of invasive disease but also plays key roles in transmission and spread of both antibiotic-susceptible and -resistant strains such as methicillin-resistant *S. aureus* (MRSA) [4–6,8–12]. Despite this significance, the host-pathogen interactions governing *S. aureus* colonization remain incompletely understood. In its human host, *S. aureus* colonizes both the anterior and respiratory epithelia of the inner nasal cavity [11,13]. Colonization of the anterior epithelium (nasal vestibule) has been modeled by measuring bacterial adhesion to human squames and inoculation of mice; these approaches revealed the contribution of the cell wall-anchored proteins ClfB, SasG, IsdA, and SdrC [14–17] in establishing interactions with host loricrin, desmoglein-1 and various keratins [18–20]. Colonization using cotton rats revealed the contribution of glycosylated wall teichoic acid (WTA), the abundant envelope polymer of *S. aureus* [21]. Glycosylated WTA was further shown to promote the interaction of bacteria with non-cornified epithelia lining the inner nasal cavity by binding to the type F scavenger receptor (SREC-I) [22–24].

Colonization of mice by *S. aureus* is transient and requires antibiotic treatment to deplete the resident microbiota [11,25,26]. Yet, spillovers to animals are not infrequent and have been shown to result in anthropogenic infection [27–29] as well as colonization [30]. We and others described mouse-adapted strains isolated from independent outbreaks of preputial gland abscesses among male C57BL/6J mice [31,32]. These strains have been shown to colonize animals for life and are passed from dams to their offspring [31,32], effectively mimicking human colonization at birth [7]. Using the mouse-adapted strain WU1, we reported earlier that an isogenic variant lacking *spa* (Δ*spa*) could not persist [32]. Failure to persist has also been reported in human participants inoculated with a *spa*-deficient mutant [33]. *spa* encodes Staphylococcal protein A (SpA), an abundant surface protein that promotes escape from complement-mediated phagocytic killing and exerts B cell superantigen activity that diverts antibody responses against the pathogen [34–40]. Indeed, decolonization of mice inoculated with Δ*spa* was found to correlate with increased serum IgG and IgA against the surface adhesins ClfB, IsdA, SasG and surface proteins SdrD, SdrE, SasI, SasD, SasA [32]. Decolonization was also observed when animals were vaccinated with two SpA immunogens, *i.e.*, modified versions of the wild type antigen that elicit neutralizing antibodies against its antiphagocytic and B cell superantigen activities, the latter correlating with increased pathogen-specific IgG and IgA responses [32,41].

Secretory IgA (SIgA) is the most abundant antibody class at mucosal surfaces and is classically considered a first line of defense against pathogens while also modulating gut microbiota composition and homeostasis [42–51]. Only a few studies have examined the interaction between SIgA in nasal secretion and bacterial pathogens and determined a role in immune exclusion [52]. Here, we use the recently described *Ighasec*^-/-^ mutant mice [53] to examine the role of SIgA in *S. aureus* colonization and SpA-based vaccine efficacy.

## Results

### Both IgA and SpA support *S. aureus* colonization

We reported earlier that mice inoculated with Δ*spa* bacteria are able to clear the pathogen from the nasopharynx possibly as the result of increased serum IgG and IgA against key surface proteins [32]. To evaluate the contribution of IgA alone in promoting such decolonization, we obtained the recently described *Ighasec*^-/-^ mutant mouse from our colleagues at the University of Chicago [53,54]. IgA deficiency was achieved by deleting the secretory tailpiece of the protein [54]. In this design, the B cells of *Ighasec*^-/-^ animals retain the ability to class switch but cannot mature into IgA plasma cells resulting in the absence of secreted IgA [53]. 16S rRNA sequencing revealed no significant difference between the relative and absolute genera abundances in bacterial communities of co-housed littermate control and *Ighasec*^-/-^ mice [53]. However, an expansion of CD8αβ^+^ intestinal intraepithelial lymphocytes that produced interferon (IFN)γ was observed in *Ighasec*^-/-^ animals and attributed to colonization by murine astrovirus [53]. The compensatory expansion of IgM and IgG plasma cells was not observed [53], unlike in the original IgA-deficient mice, that lack the entire IgA switch region and the 5’ half of the Cα region [55]. With this knowledge in mind, we proceeded to use these animals for colonization studies with *S. aureus*.

**Fig 1:**
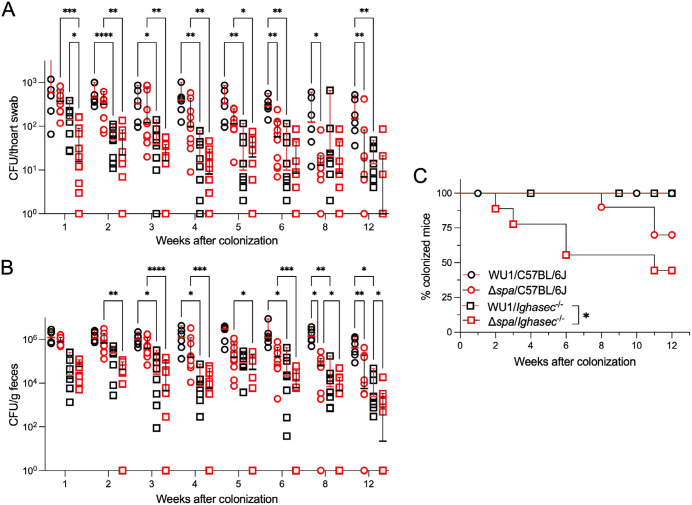
Secretory IgA promotes *S. aureus* colonization in the nasopharynx. *S. aureus* bacterial load in throat swabs **(A)** and stool samples **(B)** from C57BL/6J (circles) and *Ighasec*^-/-^ deficient mice (squares) colonized with either wild-type *S. aureus* WU1 (black symbols) or the isogenic *spa* mutant strain, Δ*spa* (red symbols). Each symbol (circle/square) represents one animal. Males and females were used at 50:50 ratio. Samples were collected over 12 weeks and bacterial burdens presented as median log₁₀ CFU and analyzed using two-way ANOVA with repeated measures. **(C)** Loss of *S. aureus* carriage in C57BL/6J and *Ighasec*^-/-^ mice shown as Kaplan-Meier curves. Loss of carriage (decolonization) was defined as the absence of detectable CFU in both swab and stool samples and the data was analyzed using the log-rank (Mantel-Cox) test.

Groups of C57BL/6J and *Ighasec*^-/-^ knockout mice were colonized with *S. aureus* WU1 (Fig 1; black circles, C57BL/6J; black squares, *Ighasec*^-/-^) or the isogenic Δ*spa* mutant (Fig 1; red circles, C57BL/6J; red squares, *Ighasec*^-/-^). Colonization was assessed by measuring bacterial burdens following plating of nasopharyngeal swabs (Fig 1A) and fecal material (Fig 1B) on mannitol salt agar (MSA), a differential and selective medium for staphylococci. The inability to culture *S. aureus* from both swabs and feces samples, was also scored as complete decolonization and the data displayed as % of animals remaining colonized over time (Fig 1C). As expected, C57BL/6J mice remained colonized with WU1 (Fig 1C), with bacterial loads in throat swabs and stool samples averaging ~2–3 log_10_ and ~5–6 log₁₀ CFU over 12 weeks, respectively (Fig 1A, 1B) [32]. These bacterial burdens match what is observed in human populations and are likely the result of the different sampling methods (collecting feces versus gentle swabs) [56]; it is also possible that *S. aureus* replicates during transit in the GI tract. Like C57BL/6J mice, *Ighasec*^-/-^ animals remained colonized with *S. aureus* (Fig 1C). Yet, WU1 burdens in throat swabs and stool samples were significantly lower starting at weeks 2 and 3 post-inoculation, respectively, as compared to C57BL/6J mice (Fig 1A, 1B; S1 Table). This result indicates that *S. aureus* may exploit IgA for optimal colonization. As observed earlier, Δ*spa* bacteria were able to colonize C57BL/6J mice but reductions in bacterial burdens were observed as early as week 6 in nasal swabs (Fig 1A; S1 Table) and week 8 in feces (Fig 1B; S1 Table), with 30% of animals fully decolonized by week 11 (Fig 1C). When Δ*spa* bacteria were inoculated in *Ighasec*^-/-^ animals, decolonization occurred even more rapidly (Fig 1C). Δ*spa* bacterial burdens were statistically reduced compared to all other groups already at weeks 1 and 2 in nasal swabs (Fig 1A; S1 Table) and fecal samples (Fig 1B; S1 Table), respectively, and 55% of animals were decolonized by week 11 (Fig 1C). Together, these observations suggest that *S. aureus* colonization is facilitated by the presence of both IgA and SpA.

**Fig 2:**
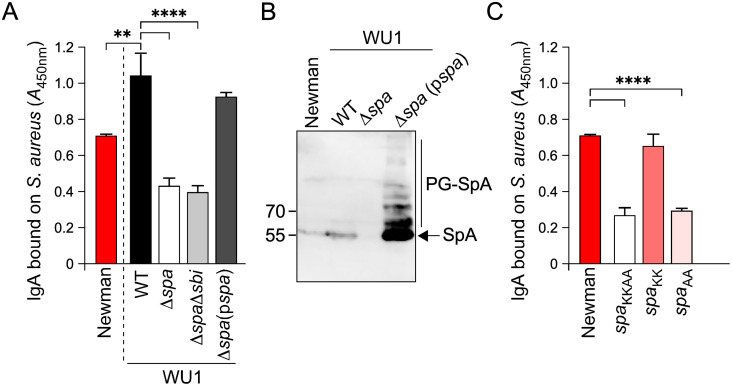
*S. aureus* binds IgA in a non-immune-dependent manner via Sp. IgA binding to *S. aureus* was measured by ELISA. **(A)** IgA binding by *S. aureus* strains, Newman, and wild type (WT) WU1, or isogenic mutants Δ*spa*, Δ*spa*Δ*sbi*, and complemented strain Δ*spa*(p*spa*). **(B)** SpA production was examined by western blot using extracts of lysostaphin-treated Newman and WU1: WT, Δ*spa*, and Δ*spa*(p*spa*) strains. The arrow points to SpA (~55 kDa) fully released from peptidoglycan (PG). The migration of the 55 and 70 kDa molecular weight markers is indicated to the left of the blot. SpA is over produced in the complemented strain and not all SpA molecules were efficiently released from PG accounting for the additional bands noted as PG-SpA. A representative blot is shown. **(C)** IgA binding by Newman and the following isogenic variants, *spa*_KK_ (Fc-binding disrupted, Fab-V_H_3 binding intact), *spa*_AA_ (Fc-binding intact, Fab-V_H_3 binding disrupted), and *spa*_KKAA_ (both binding sites disrupted). Differences were analyzed with one-way ANOVA (A, C).

We reasoned that the pathogen could exploit IgA through direct interaction with surface exposed SpA. Indeed, SpA encompasses five Immunoglobulin-binding domains and each module can bind to the Fcγ-domain of mouse IgG1 and IgG2a/c and human IgG1, IgG2, IgG4, as well as to the variant heavy chains of V_H_3-like clonal IgM, IgG, IgE, IgD and IgA (the numbers of the variable heavy chain regions in humans and mice do not concord; here, V_H_3 is used loosely to designate the ligand of SpA in both species) [35,57–63]. To test this possibility, bacteria were adsorbed to 96-well plates and mouse IgA binding assessed in an indirect Enzyme-Linked Immunosorbent Assay (ELISA). Plates were coated with *S. aureus* Newman, WU1, WU1 Δ*spa*, WU1 Δ*spa* Δ*sbi* and WU1 Δ*spa*(p*spa*) (WU1 Δ*spa* complemented with plasmid encoded *spa*) (Fig 2A). The double mutant WU1 Δ*spa* Δ*sbi* was included as Sbi binds the Fcγ-domain of IgG [64] (Fig 2A, 2B). To further investigate Ig-Fc versus Fab-V_H_3 binding, we used strain Newman, and its isogenic variants *spa*_KK_, *spa*_AA_, and *spa*_KKAA_ (Fig 2C), respectively [36]. The experiment was performed in this manner because *spa*_KK_, *spa*_AA_, and *spa*_KKAA_ variants have not been generated in strain WU1. *spa*_KK_ bacteria bind Fab-V_H_3 but not the constant region of IgG (Ig-Fc), *spa*_AA_ cells bind Ig-Fc but not Fab-V_H_3, and *spa*_KKAA_ cells bind neither Ig-Fc nor Fab-V_H_3 [36]. IgA interacted with both WU1 and Newman and binding was slightly higher with strain WU1 (Fig 2A; S2 Table). In strain WU1, most of this interaction was *spa*-dependent and *sbi*-independent and was complemented upon plasmid expression of *spa* (Fig 2A; S2 Table). When using Newman variants, it became obvious that IgA associates with SpA in a Fab-V_H_3-dependent manner (Fig 2A; S2 Table). A western blot analysis revealed that Newman produces slightly less SpA compared to WU1 (Fig 2B). This analysis also documented plasmid complementation in strain WU1 Δ*spa*(p*spa*), although increased IgA production over wild type did not result in a measurable increase in IgA binding by ELISA (Fig 2A, 2B; WU1 Δ*spa*(p*spa*) versus WU1). We conclude that during colonization, *S. aureus* bacteria could be coated with IgA-V_H_3 antibodies in a non-immune-dependent manner via SpA. Together, these results support the hypothesis that the pathogen exploits IgA to promote its colonization.

### Distinct antibody isotype patterns are observed during colonization in absence of SpA or IgA

**Fig 3:**
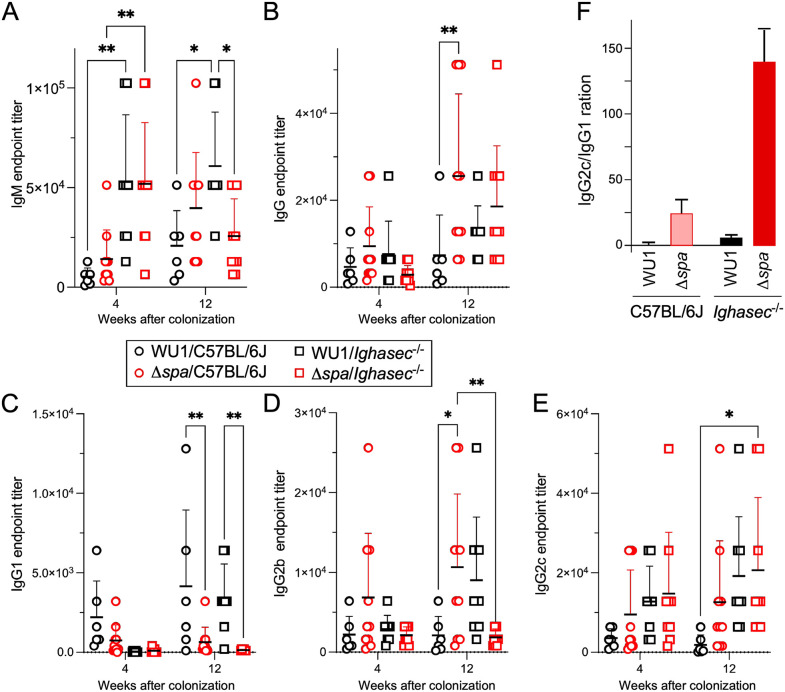
Antibody responses against *S. aureus* during the course of colonization. Animals shown in Fig 1 were bled at weeks 4 and 12 post colonization to measure antibody responses against *S. aureus* using plates coated with a crude extract of Δ*spa*Δ*sbi* bacteria. Data are shown as endpoint titers of serum IgM **(A)**, total IgG **(B)**, IgG1 **(C)**, IgG2b **(D)**, and IgG2c **(E)** specific to *S. aureus*. Differences were analyzed using a two-way ANOVA with post hoc tests. **(F)** IgG subclass polarization shown as the IgG2c/IgG1 ratio.

We wondered if antibody responses to *S. aureus* are altered during successful decolonization. To assess this possibility, animals shown in Fig 1 were bled at weeks 4 and 12 post-colonization and sera were used to quantify the amounts of IgM, total IgG, as well as IgG1, IgG2b, and IgG2c directed against *S. aureus* (Fig 3A-3E; S3 Table). Endpoints titers were determined after coating plates with normalized cellular extracts of *S. aureus* lacking both immunoglobulin binding proteins (WU1Δ*spa*Δ*sbi*), and data compared by collection times (either 4 or 12 weeks post colonization). At week 4 post-inoculation with bacteria, *Ighasec*^-/-^ mice exhibited significantly higher IgM titers against *S. aureus* than C57BL/6J animals in a *spa*-independent manner (Fig 3A; S3 Table), which could account for the lower bacterial burden observed in these animals (Fig 1). IgM levels in all groups was sustained at week 12, indicating that IgM production is primarily driven by antigenic stimulation. An analysis of total IgG responses shows that at week 12 post inoculation, C57BL/6J mice colonized with Δ*spa* exhibited significantly higher anti-*S. aureus* IgG responses than animals colonized with WU1, in agreement with the notion that immune recognition of bacterial antigens is ameliorated in the absence of SpA (Fig 3B) [36,38,39]. No other differences were noticed between the remaining groups (S3 Table). Deconvolution of isotype-specific anti-*S. aureus* IgG responses revealed that in absence of SpA, colonized C57BL/6J mice preferentially develop IgG2b and IgG2c over IgG1 responses (Fig 3C-3E). Colonization of *Ighasec*^-/-^ mice with Δ*spa* resulted in similarly skewed IgG responses with an even greater reduction of IgG1 at weeks 4 and 12 (Fig 3C-3E). When displayed as an IgG2c/IgG1 ratio (Fig 3F), these differences are striking. Studies in mice established that secretion of IgG1 antibodies by IL-4-regulated B cells and production of IgG2a/c antibodies upon IFNγ stimulation are direct indicators of the underlying Th2 or Th1 response with lower IgG2c/IgG1 values reflecting a Th2 response while higher values indicating a Th1 response [65,66]. *Ighasec*^-/-^ mice colonized with Δ*spa* exhibit the highest IgG2c/IgG1 ratio (~140, Fig 3F), and the highest bacterial clearance (Fig 1). C57BL/6J mice colonized with Δ*spa* have a lower IgG2c/IgG1 ratio (~25, Fig 3F) and display moderate decolonization over the same observation period (Fig 1). In contrast, WU1-colonized C57BL/6J animals that remain persistently colonized display the lowest IgG2c/IgG1 ratio (~0.3, Fig 3F). In WU1-colonized *Ighasec*^-/-^ mice, this ratio is shifted toward a Th1 response (~4, Fig 3F). This shift may explain the reduced bacterial burdens (Fig 1). Together, these findings reveal that decolonization is associated with a strong Th1 response against the pathogen. Expression of *spa* either dampens the Th1 response or promotes a Th2 response to favor bacterial persistence. Although animals lacking IgA develop a Th1 response during colonization, this response is not sufficient to overcome SpA-mediated immune evasion.

### Antigen-specific responses to *S. aureus* in absence of SpA or IgA

**Fig 4:**
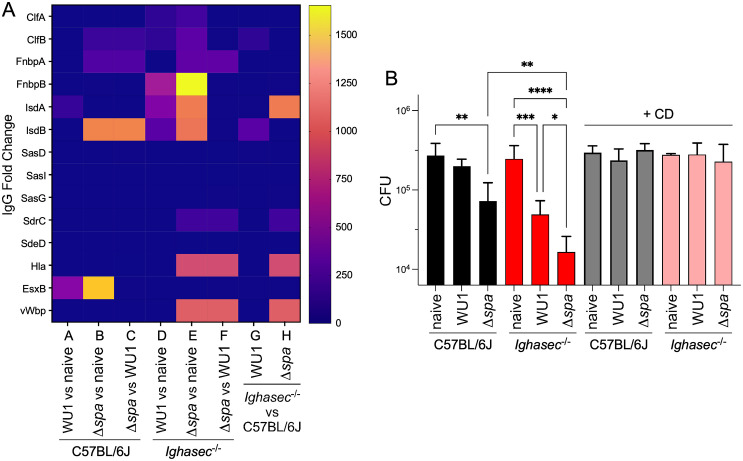
Breadth and protective nature of antibody responses in C57BL/6J and *Ighasec*^-/-^ animals colonized with WU1 orΔ*spa* bacteria. **(A)** Week 12 sera samples of animals shown in Fig 1 were assessed for the presence of antibody against 14 *S. aureus* antigens (names are listed on the left side of the panel). IgG fold changes between animal groups were captured as a heatmap with intensity scale shown to the right of the panel. Groups A–F represent IgG responses between C57BL/6J and *Ighasec*^-/-^ animals, while Groups G and H compare IgG responses between C57BL/6J and *Ighasec*^-/-^ mice colonized with the same strain. A: C57BL/6J mice colonized with WU1 vs. naive C57BL/6J; B: C57BL/6J mice colonized with Δ*spa* vs. naive C57BL/6J; C: C57BL/6J colonized with Δ*spa* vs. WU1; D: *Ighasec*^-/-^ mice colonized with WU1 vs. naive *Ighasec*^-/-^; E: *Ighasec*^-/-^ mice colonized with Δ*spa* vs. naive *Ighasec*^-/-^; F: *Ighasec*^-/-^ mice colonized with Δspa vs. WU1; G: *Ighasec*^-/-^ vs. C57BL/6J mice colonized with WU1; H: *Ighasec*^-/-^ vs. C57BL/6J mice colonized with Δspa. **(B)** Opsonophagocytic killing activity of the same sera analyzed in panel A was assessed by measuring *S. aureus* replication in freshly drawn blood of B6J.μMT mice. Red bars depict samples that receive the cytochalasin D (CD) control, confirming that bacterial killing was phagocytosis-dependent. The data was analyzed with one-way ANOVA.

In the above section, crude bacterial extracts were probed to examine antibody responses against *S. aureus.* Sera collected prior to colonization (naïve controls) or at week 12 post-inoculation were also examined for reactivity toward fourteen secreted antigens of *S. aureus*, four of which, ClfB, IsdA, SasG, and SdrC, represent *bone fide* colonization factors [14–17]. IgG fold changes to the various antigens were normalized to immune reactivity in naïve sera and depicted as a heatmap (Fig 4A). C57BL/6J mice colonized with WU1 exhibited low IgG responses across most antigens, with a modest recognition of IsdA and EsxB, conforming with the notion that colonization with wild-type *S. aureus* does not elicit strong systemic IgG responses (Fig 4A; Group A). In contrast, IgG levels against ClfB, FnbpA, IsdB, and EsxB, were increased in C57BL/6J animals colonized with Δ*spa* compared to naïve animals (Fig 4A; Group B) and IgG levels against three of these antigens (ClfB, FnbpA, IsdB) were higher when compared to WU1 (Fig 4A; Group C). When the same analysis was performed for *Ighasec*^-/-^ mice, a wider magnitude and breadth of IgG responses were observed (Fig 4A; Groups D-F). Colonization of *Ighasec*^-/-^ animals with WU1 elicited a more varied IgG response notably against ClfA, ClfB, FnbpB, IsdA and IsdB (Fig 4A; Group D) compared to the muted response in C57BL/6J mice (Fig 4A; Group A). IgG fold changes between naïve and colonized mice were even more obvious in *Ighasec*^-/-^ animals colonized with Δ*spa* (Fig 4A; Group E) with increased reactivities to multiple antigens, including ClfA, ClfB, FnbpA, FnbpB, IsdA, IsdB, SdrC, Hla, and vWbp. Thus, SpA dampens host immune responses in both C57BL/6J and *Ighasec*^-/-^ animals. But absence of IgA also results in the derepression of antibody responses as noted by the increased IgG levels against ClfB and IsdB in animals colonized with WU1 (Fig 4A; Group G) and against IsdA, SrdC, Hla and vWbp in animals colonized with Δ*spa* (Fig 4A; Group H). The strongest antibody responses occurred when both SpA and IgA were absent correlating with the efficient decolonization rate observed in *Ighasec*^-/-^ animals colonized with Δ*spa* (Fig 1).

While we do not yet know how anti-*S. aureus* antibodies promote decolonization, we can assess the opsonophagocytic activity of such antibodies using a whole blood killing assay [67,68]. Sera from WU1 and Δ*spa* colonized C57BL/6J and *Ighasec*^-/-^ animals (shown in Fig 1) as well as sera from naïve mice (controls) were added to anti-coagulated, freshly drawn blood of naïve B6J.μMT mice and then incubated with *S*. *aureus*. B6J.μMT mice lack most immunoglobulins owing to their immature B cells, but provide the phagocytes needed to assess the activity of test sera [69,70]. To confirm that bacterial killing is phagocyte-dependent, cytochalasin D (CD), an inhibitor of actin polymerization and thus phagocytosis, was added to a duplicated set of samples. Following 30 minutes incubation with bacteria, samples were treated with saponin, streptokinase, and ribonuclease, to liberate intracellular bacteria from host cells and extracellular bacteria in fibrin agglutinates and in neutrophil extracellular traps, respectively [67]. Surviving bacteria were enumerated following plating (Fig 4B; S4 Table). Serum from C57BL/6J mice colonized with WU1 did not show any reduction in bacterial replication compared to serum from naïve animals. In contrast, serum from C57BL/6J mice colonized with Δ*spa* exhibited significantly enhanced opsonophagocytic killing activity (Fig 4B; S4 Table). Serum from colonized, but not naïve, *Ighasec*^-/-^ mice also exhibited significant opsonophagocytic killing activity, particularly animals that had been colonized with Δ*spa* (Fig 4B). All killing activities were abrogated in the presence of CD (Fig 4B). Thus, increases in IgG titers against multiple *S. aureus* antigens and in IgG2c over IgG1 correlate with increased opsonophagocytic killing activity and decolonization.

### IgA deficiency enhances vaccine-mediated clearance of *S. aureus*

**Fig 5:**
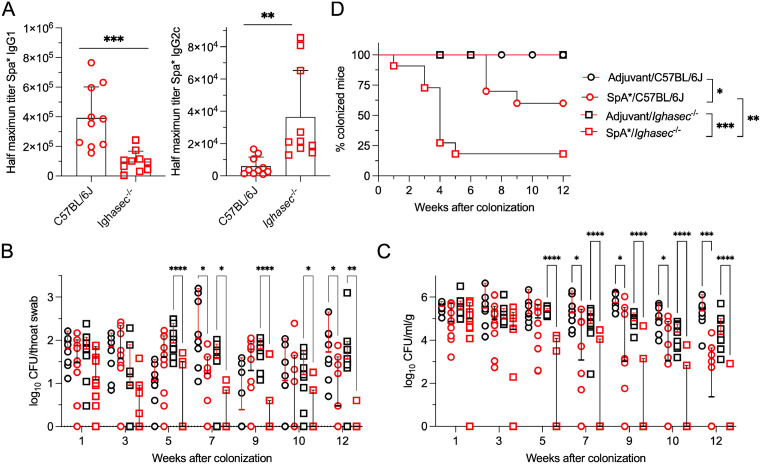
Vaccine-mediated clearance of *S. aureus* is enhanced in *Ighasec*^-/-^ animals. Animals were vaccinated with the SpA* antigen (red symbols) or adjuvant alone (black symbols) and colonized as in Fig 1. **(A)** Serum antibody responses in animals vaccinated with SpA*. Left: total IgG titers; right: IgG2c titers. Data were analyzed with the unpaired *t*-test. All data represent median values with interquartile ranges. **(B)** Swabs and **(C)** stool samples from each animal were plated on MSA to enumerate bacterial burdens and the data presented as median log₁₀ CFU following by two-way ANOVA with repeated measures. Each symbol (circle/square) represents one animal. Males and females were used at 50:50 ratio. **(D)** Loss of *S. aureus* carriage in C57BL/6J and *Ighasec*^-/-^ mice shown as Kaplan-Meier curves. Loss of carriage (decolonization) was defined as the absence of detectable CFU in both swab and stool samples and the data was analyzed using the log-rank (Mantel-Cox) test.

We reported earlier that vaccination with SpA_KKAA_ or SpA_KKE_ (herein referred as SpA*), two immunogens that carry the five Ig-binding domains of SpA with amino acid substitutions that abolish the ability to bind Fcγ or Fab V_H_3, elicit neutralizing antibodies against SpA [41,71]. Such immunizations promote the decolonization of *S. aureus* from the nasopharynx of mice in a manner that correlates with the development of both anti-SpA and broad anti-*S. aureus* IgG and IgA antibodies [32,41]. To assess whether IgA deficiency may affect vaccine-induced clearance of colonizing *S. aureus*, groups of C57BL/6J and *Ighasec*^-/-^ mice were immunized with adjuvant alone (mock) or SpA* (Fig 5; S5 Table). Complete Freund Adjuvant was used to conform with prior studies [32,41]. Serum antibody responses against SpA* were assessed by ELISA prior to intranasal inoculation with *S. aureus* WU1 (Fig 5A). Total anti-SpA* IgG titers were similar in both C57BL/6J and *Ighasec*^-/-^ animals; yet, anti-SpA* IgG1 titers were approximately 3.9 times higher in C57BL/6J compared to *Ighasec-/-* mice while conversely anti-SpA* IgG2c titers were 6 times higher in *Ighasec*^-/-^ compared to C57BL/6J animals (Fig 5A; S5 Table). Despite these altered ratios, IgG1 remained the dominant subclass in both animal strains. As expected, compared to mock treatment, Spa* immunization resulted in reduced bacterial burdens in C57BL/6J animals as soon as week 7 post-inoculation (Fig 5B, 5C; S5 Table), with complete decolonization of 40% of animals by week 9 (Fig 5D). Interestingly, the efficacy of the vaccine was fully retained in animals lacking IgA (Fig 5B-5D). In fact, decolonization was even more rapid, beginning as early as week 1 post-inoculation in *Ighasec*^-/-^ vaccinated mice with 80% of animals fully decolonized by week 5 (Fig 5B, 5C; S5 Table). Adjuvant-treated *Ighasec*^-/-^ mice remained colonized over the entire observation period, suggesting that decolonization is specific to SpA* vaccination (Fig 5D). The reduction in bacterial burdens observed in *Ighasec*^-/-^ mice compared to C57BL/6J animals (Fig 1A, 1B), was lost upon treatment of animals with CFA/IFA adjuvant (Fig 5B, 5C). Together, these results indicate that IgA is dispensable for SpA* vaccine-mediated decolonization, at least under the experimental conditions tested. The vaccine worked even better in *Ighasec*^-/-^ animals compared to wild type C57BL/6J mice, which could be the result of increased anti-SpA* IgG2c titers.

## Discussion

In human and mouse, IgA is the most abundant antibody and, in the gut lumen where its function has been extensively examined, SIgA facilitates immune exclusion through a range of activities that help limit access of intestinal antigens to the blood, neutralize pathogens and toxins, and regulate inflammation at epithelial surfaces [44]. SIgA also coats bacteria in a manner that helps shape a healthy and diverse gut microbiota [42,43,46–50]. Conversely some gut pathogens such as *Escherichia coli* or *Helicobacter pylori* exploit SIgA to form biofilms [72] and persist in the gastric mucosa [73,74].

**Fig 6:**
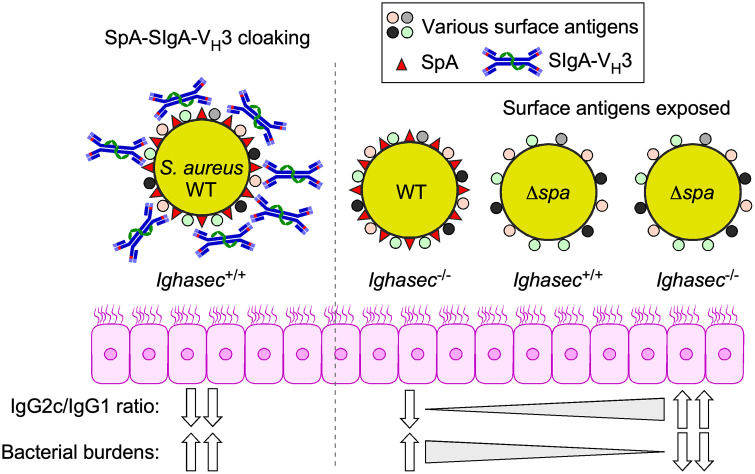
IgA contributes to *S. aureus* colonization: A model. During colonization, surface exposed SpA engages Secretory (S)IgA-V_H_3 antibodies at the nasal mucosa to generate a non-opsonic immune cloak. Because murine SIgA does not activate the classical complement pathway and mice lack FcαRI, the SpA–SIgA coating effectively masks bacterial surface epitopes, limiting access by opsonic antibodies and blunting the development of high-affinity responses (cloaking). SpA may also function as an antibody decoy to reduce the pool of functional IgA. In the absence of SpA or SIgA, surface epitopes of *S. aureus* become accessible and a Th1-skewed IgG2c response that favors bacterial clearance is observed. How is isotype switching modulated by SpA is unknown. In the case of IgA deficient animals, skewing may be the result of persistent viral colonizers in the gut that elicit an IFNγ response. WT: wild type.

The nasal microbial community in humans is not as abundant or diverse as the gut microbiome [75–77]. The upper respiratory tract is also the site of colonization of human-adapted pathogens including, *Streptococcus pneumoniae*, *Neisseria meningitidis*, *Haemophilus influenzae* and *S. aureus.* Colonization by these pathogens occurs seemingly in a non-inflammatory state and represents a key predisposing risk factor for invasive disease [9,78–80]. A number of nasopharyngeal and oral cavity pathogens, including the aforementioned *S. pneumoniae*, *N. meningitidis*, and *H. influenzae* have the potential to abrogate the protective activity of SIgA by producing human IgA1-specific proteases [81,82]. Here we sought to examine how *S. aureus* engages with SIgA by colonizing wild type and mutant mice lacking IgA. Our findings suggests that SIgA facilitates *S. aureus* persistence in the nasopharynx possibly via two mechanisms (Fig 6). A first mechanism involves SpA binding to V_H_3-Ig including V_H_3-IgA [35,36]. SpA binding to Fab V_H_3-B cell receptors exerts a B cell superantigen activity that skews antibody responses away from the pathogen and triggers the production of non-specific V_H_3-rearranged antibodies [36,38,39]. We surmise that such dilutive antibodies include V_H_3-SIgA and bind surface exposed SpA on the pathogen. V_H_3-SIgA coating of *S. aureus* may limit the accessibility of antigenic epitopes for IgG binding that would otherwise promote opsonization or complement activation (Fig 6; cloaking mechanism). This proposed mechanism would agree with a recent study showing that *S. aureus* binds SIgA in filtered human nasal eluates in a SpA-dependent manner [77]. Importantly, in this study, high SIgA binding to *S. aureus* did not correlate with lower bacterial carriage [77]. This was unlike with other nasal colonizers for which SIgA abundance in nasal eluates correlated with low bacterial density pointing to the canonical protective role of SIgA [77].

We also observe that *Ighasec*^-/-^ mice develop slightly higher levels of anti-*S. aureus* IgG2c antibodies as compared to wild type mice (Fig 6; IgG2c shift). This shift in isotypes upon colonization may reflect an inherent phenotype of IgA-deficiency or a broader role for SIgA in shaping mucosal immune responses. Our colleagues who generated the *Ighasec*^-/-^ mouse reported that loss of SIgA does not impact gut bacterial communities but correlates with murine astrovirus colonization which in turn stimulates the expansion of IFNγ-producing cells in the small intestine [53]*.* This increased interferon response while not sufficient to clear the virus, limits viral loads and adverse immune responses [53]. Since *Ighasec*^-/-^ mice transferred to our colony retained the astrovirus, the sustained IFNγ production in the gut could explain the reduction in bacterial burdens observed in Fig 1; but as with the virus, this response was not sufficient to promote decolonization. In the gastrointestinal tract, SIgA has also been shown to bind some commensal bacteria and promote their uptake by Peyer’s patch dendritic cells resulting in the induction of T regulatory cells with suppressive function [50,83–85]. Whether *S. aureus* is similarly internalized during colonization in a V_H_3-IgA and SpA-dependent manner, remains to be determined. But, enhanced IgG2c antibody responses that correlate with the loss of *S. aureus* carriage, are also observed when animals are colonized with *S. aureus* lacking *spa* (Fig 6). *Ighasec*^-/-^ animals colonized withΔ*spa S. aureus* develop both a broader polyclonal response to several bacterial antigens (in absence of the B cell superantigen effect of SpA) and increased production of IgG2c over IgG1 antibodies (Fig 6). We do not yet know the molecular mechanism underlying the SpA-mediated shift in isotypes, but these observations suggest that serum antibodies, in absence of SIgA, can eliminate *S. aureus* from the nasopharynx of colonized animals. This shift is also reflective of a favorable Th1 response. Although we have yet to examine cytokine production in these animals, it is noteworthy that exaggerated Th2 responses are observed in conditions like atopic dermatitis where barrier integrity and local immune defenses are altered and correlate with *S. aureus* colonization [86,87].

The effectiveness of the SpA* vaccine is also enhanced in *Ighasec*^-/-^ animals. While Spa* vaccination induced anti-Spa IgG responses in both wild type and *Ighasec*^-/-^ mice, bacterial clearance was most effective in mutant animals. We presume that the same mechanisms described above are also at play in a vaccination that aim to neutralize the immunosuppressive attributes of SpA. In absence of IgA, the development of serum antibodies is thus sufficient to clear the pathogen. This is not unlike the findings that prevention of invasive diseases by *S. pneumoniae*, *N. meningitidis*, or *H. influenzae* by licensed conjugate vaccines correlates with the development of serum opsonophagocytic and bactericidal antibodies that also reduce nasopharyngeal colonization and reduce host-to-host transmission [88–94].

While our murine model effectively recapitulates key aspects of *S. aureus* colonization, certain limitations must be acknowledged. In humans, monomeric IgA immune complexes (but not polymeric SIgA) can bind to and activate the high-affinity IgA receptor, FcαRI, displayed on granulocytes, monocytes, macrophages, DCs, natural killer cells, and mast cells [95]. A functional homologue of FcαRI in mice has not been identified [95]. Further, mice produce a single IgA class, while human B cells produce IgA1 (both systemic and mucosal), and IgA2, a protease-resistant subclass found in microbiota-rich mucosa such as the intestinal and urogenital tracts [95]. Future studies should further explore SIgA-SpA interactions, the impact of SpA on isotype switching, as well as the impact of IgA-specific antibodies against the pathogen. Indeed, current immunization approaches against nasopharyngeal pathogens (*S. pneumoniae*, *N. meningitidis*, or *H. influenzae*), including our own experiment, used parenteral modes of administration. We have yet to examine if strategies favoring the elicitation of neutralizing IgA antibodies might further improve the mucosal clearance of the pathogen.

## Materials and methods

### Ethics statement

Animal research was performed in accordance with institutional guidelines following experimental protocol review, approval, and supervision by the Institutional Animal Care and Use Committee at The University of Chicago (protocol approval number 71022). Experiments with *S. aureus* were performed in Biosafety Level 2 containment upon review by The University of Chicago Institutional Biosafety Committee.

### Bacterial strains and growth conditions

*S. aureus* strains Newman and WU1 and isogenic variants lacking *spa* or *sbi* (∆*spa* and ∆*spa*∆*sbi*) or expressing *spa*_AA_, *spa*_KK_, *spa*_KKAA_, or plasmid encoded *spa* (p*spa*) were from our laboratory collection. Strains were routinely cultured in tryptic soy broth (TSB) or on tryptic soy agar (TSA) plates at 37°C. Animal swabs and fecal samples, from colonized mice, were plated on mannitol salt agar (MSA) and incubated at 37°C for bacterial enumeration.

### Preparation of bacterial cultures and extracts

Briefly, bacterial inocula used for animal colonization and whole blood killing assays were prepared as follows. A single colony from an agar plate was used to inoculate TSB for an overnight culture which was subsequently diluted 1:100 into fresh TSB and grown until the culture reached an optical density at 600 nm (OD_600_) of approximately 1. Bacterial cells were collected by centrifugation, washed and resuspended in 25 mL PBS. After a second centrifugation, the cells were resuspended in PBS to a final concentration of ~1 × 10¹⁰ CFU/mL. Bacterial suspensions were used immediately. 10 µL of this suspension (~1 × 10⁸ CFU) was used for intranasal inoculation of animals. For ELISA and western blot experiments shown in Fig 2, bacterial cultures were grown in the same manner, washed, and normalized to an OD_600_ of 1. For ELISA experiments that measure serum antibody titers against *S. aureus* (Figs 3A-3E and 5A), the cell suspensions normalized to an OD_600_ of 1, were further treated with 20 µg/mL lysostaphin for 1h at 37°C before sonication on ice for 30 s. These crude extracts were stored at -80**°**C until use.

### Animal experiments

C57BL/6J (stock #000664) and B6J.μMT (B6.129S2-*Ighm*^*tm1Cgn*^/J, stock 002288) mice were purchased from The Jackson Laboratory. *Ighasec*^-/-^ mice were a gift from Dr. Albert Bendelac (The University of Chicago) [53]. All animals were bred at the University of Chicago. Six to eight-week-old animals (50% female, 50% male) were used in all experiments. For intranasal inoculation and blood draw, animals were anesthetized with a cocktail of ketamine-xylazine (50–65 and 3–6 mg/kg). For immunization studies, animals received either PBS or 50 µg of endotoxin-free Spa* immunogen emulsified in a 5:2:3 ratio of antigen:CFA:IFA for the initial immunization. A booster dose of 50 µg Spa*, emulsified in a 1:1 ratio of antigen:IFA, was given 11 days later. On day 20, serum was collected via submandibular bleeding to assess antibody titers by ELISA. On day 21, mice were inoculated intranasally for nasopharyngeal colonization as described [32]. Following intranasal inoculation with bacteria, mice were monitored daily and throat swabs and fecal pellets were obtained in weekly intervals. Fecal pellets were collected into sterile pre-weighed tubes, weighed, and homogenized in 500 μl of PBS before serial dilution plating while throat swabs were plated directly [32].

### Whole blood killing assay

Freshly collected blood (300 µL) from B6J.μMT mice, anticoagulated with heparin (10 U/mL), was used for the whole blood-killing assay. Blood samples were pre-incubated for 15 minutes at 37°C with 20 µM cytochalasin D dissolved in DMSO or with DMSO alone as the vehicle control. After pre-incubation, 15 µL of pooled serum obtained from either naïve or colonized mice was added to each tube. To initiate the reaction, 15 µL of PBS containing 1.0 × 10⁴ CFU of *S. aureus* was added, and samples were incubated for 30 minutes at 37°C with gentle rotation. Following incubation, 300 µL of SK buffer (containing 2% saponin, 200 U/mL streptokinase, 1 mg/mL trypsin, 20 µg/mL DNase, and 100 µg/mL RNase A) was added to each sample and incubated for an additional 10 minutes at 37°C to disaggregate any bacterial clumps [67]. After treatment, samples were plated to determine CFU counts. The experiment was repeated independently once, and all assays were conducted in duplicate.

### Enzyme-Linked Immunosorbent Assay (ELISA)

For ELISA experiments that measure IgA binding to bacteria (Fig 2A and 2C), microtiter plates (NUNC MaxiSorp) were coated with *S. aureus* WU1, WU1 Δ*spa,* WU1 ∆*spa*∆*sbi,* WU1 Δ*spa* (p*spa*), Newman or Newman derivatives expressing modified Spa variants in PBS and incubated overnight at 4°C. Plates were blocked with 5% BSA in PBS-T (PBS containing 0.05% Tween-20) before adding two-fold serial dilutions of IgA at room temperature for 1 hour. Bound IgA was detected using a biotinylated anti-IgA antibody followed by avidin-HRP, followed by the addition of TMB substrate. Absorbances were measured at 450 nm (*A*_450_) using a microplate reader. For ELISA experiments that measure antibody responses (Figs 3 A-3E; 5A), microtiter plates were coated with 0.5 μg/ml of ∆*spa*/∆*sbi* bacterial extracts as prepared above or 1 µg/mL of purified SpA* in 0.1 M carbonate buffer (pH 9.5) at 4°C overnight. Wells coated with ∆*spa*∆*sbi* bacterial extracts (Fig 3 A-3E) were blocked with 2% BSA and 5% normal goat serum in PBST. Wells coated with SpA* (Fig 5A) were blocked with 3% BSA in PBST). Serum samples were added in two-fold serial dilutions for total anti-*S. aureus* antibodies and five-fold serial dilutions for anti-Spa* antibodies and incubated at room temperature for 1 hour. Bound antibodies were detected with goat-derived biotinylated secondary antibody and avidin-HRP and signal developed using TMB substrate. IgG2c/IgG1 ratios derived from these experiments (as shown in Fig 3F) were calculated using the endpoint titer value for each serum. Endpoint titers were defined as the highest serum dilution that yielded an absorbance value (A₄₅₀) greater than the mean plus three standard deviations of the absorbance values from wells containing no serum (blank control). The IgG2c/IgG1 ratio was then calculated as (Endpoint Titer IgG2c)/(Endpoint Titer IgG1). All experiments were performed in triplicate to calculate averages and standard error of the mean and repeated for reproducibility.

### Staphylococcal antigen matrix

Antibody responses against specific *S. aureus* antigens were analyzed using a staphylococcal antigen matrix assay, as previously described [32]. Briefly, 2 µg of affinity-purified staphylococcal antigens were blotted onto nitrocellulose membranes and blocked with 5% degranulated milk to prevent non-specific binding. The membranes were then incubated with mouse sera (1:10,000 dilution), followed by IRDye 680-conjugated goat anti-mouse IgG (Li-Cor) as the detection antibody. Fluorescent signal intensities were recorded using the Odyssey infrared imaging system (Li-Cor) and quantified as fold-changes shown in a heatmap (Fig 4A). For each antigen, fold-change in fluorescent signal intensities were computed using the median signal intensity of the indicated cohort divided by the median of the comparator cohort.

### Statistical analyses

All experiments were performed at least twice. For experiments with repeated measures, data was plotted as median ± 95% confidence interval and analyzed using two-way analysis of variance (ANOVA) with Tukey’s multiple-comparison tests (GraphPad Software). Bacterial survival in blood and IgA binding data, were evaluated using one-way ANOVA followed by Tukey’s post hoc multiple comparisons test. The Mantel-Cox test was used to analyze the complete loss of *S. aureus* carriage in animals. Antibody responses were presented as mean ± SD and analyzed using two-way repeated measures ANOVA followed by Tukey’s multiple comparisons test.

## Supporting information

S1 Table Raw data and statistical analyses for experiments shown in Fig 1. Weekly bacterial loads expressed as log_10_ CFU in nasal swabs and log_10_ CFU per gram in feces for WU1 and Δ*spa* strains in C57BL/6J and *Ighsec*^−/−^ mice. Data are presented as medians with lower and upper limits corresponding to the 25th and 75th percentiles (interquartile range).
(XLSX)

S2 Table Raw data and statistical analyses for experiments shown in Fig 2. Mean ± SD of IgA binding to *S. aureus* strains measured by ELISA. (XLSX)

S3 Table Raw data and statistical analyses for experiments shown in Fig 3. Serum immunoglobulin levels (IgM, IgG, IgG1, IgG2b, IgG2c; mean ± SD) measured at 4 and 12 weeks after infection with *S. aureus* WU1 or the isogenic Δ*spa* mutant in C57BL/6J and IgA-deficient (*Ighasec*^−/−^) mice. (XLSX)

S4 Table Raw data and statistical analyses for experiments shown in Fig 4. Mean ± SD values of *S. aureus* survival in freshly drawn blood from B6J.µMT mice incubated with sera from C57BL/6J or IgA-deficient (*Ighasec*^-/-^) mice, with or without cytochalasin D (CD). (XLSX)

S5 Table Raw data and statistical analyses for experiments shown in Fig 5. Bacterial colonization and serum antibody responses following intranasal immunization with SpA* or adjuvant control in C57BL/6J and IgA-deficient (*Ighasec*^-/-^) mice. Median *S. aureus* CFU (log_10_).
(XLSX)
